# Patient-Derived iPSCs and iNs—Shedding New Light on the Cellular Etiology of Neurodegenerative Diseases

**DOI:** 10.3390/cells7050038

**Published:** 2018-05-08

**Authors:** Bor Luen Tang

**Affiliations:** 1 Department of Biochemistry, Yong Loo Lin School of Medicine, National University of Singapore, Singapore 117597, Singapore; bchtbl@nus.edu.sg; Tel.: +65-6516-1040; 2 NUS Graduate School for Integrative Sciences and Engineering, National University of Singapore, Singapore 117597, Singapore

**Keywords:** induced pluripotent stem cells (iPSCs), induced neuronal (iN) cells, C9ORF72, Huntingtin, amyotrophic lateral sclerosis (ALS), Huntington’s disease, neurodegenerative diseases

## Abstract

Induced pluripotent stem cells (iPSCs) and induced neuronal (iN) cells are very much touted in terms of their potential promises in therapeutics. However, from a more fundamental perspective, iPSCs and iNs are invaluable tools for the postnatal generation of specific diseased cell types from patients, which may offer insights into disease etiology that are otherwise unobtainable with available animal or human proxies. There are two good recent examples of such important insights with diseased neurons derived via either the iPSC or iN approaches. In one, induced motor neurons (iMNs) derived from iPSCs of Amyotrophic lateral sclerosis/Frontotemporal dementia (ALS/FTD) patients with a *C9orf72* repeat expansion revealed a haploinsufficiency of protein function resulting from the intronic expansion and deficiencies in motor neuron vesicular trafficking and lysosomal biogenesis that were not previously obvious in knockout mouse models. In another, striatal medium spinal neurons (MSNs) derived *directly* from fibroblasts of Huntington’s disease (HD) patients recapitulated age-associated disease signatures of mutant Huntingtin (mHTT) aggregation and neurodegeneration that were not prominent in neurons differentiated indirectly via iPSCs from HD patients. These results attest to the tremendous potential for pathologically accurate and mechanistically revealing disease modelling with advances in the derivation of iPSCs and iNs.

## 1. From iPSCs to iNs—Advances in the Generation of Specific Neuron Types from Patient-Derived Cells

The loss of irreplaceable adult central nervous system (CNS) neuron types underlies the state of devastating morbidity in neurodegenerative disorders like Alzheimer’s disease (AD), Parkinson’s disease (PD), Amyotrophic lateral sclerosis/Frontotemporal dementia (ALS/FTD) and Huntington’s disease (HD). These neurodegenerative diseases are all age-associated, largely sporadic (with the exception of HD) and have no effective treatment. Drug discovery and studies on disease etiology and pathological mechanisms are hampered by a lack of disease models that closely mimic human neurons and the human diseased brain. The last decade of advances in human somatic cell reprogramming has, however, drastically transformed the research landscape of neurodegenerative diseases. In particular, the ability to reprogram differentiated adult cells that could be harvested via relatively simple biopsy procedures, has effectively allowed the generation of human neurons [1] and lately brain organoids [2], from diseased patients for laboratory investigations.

A breakthrough in somatic cell reprogramming occurred in 2007, when Yamanaka and colleagues reported the generation of induced pluripotent stem cells (iPSCs) from adult human fibroblasts via the co-expression of four transcription factors, Oct3/4, Sox2, Klf4, and c-Myc [3]. The pluripotency of cells generated by this method is attested by the fact that mouse iPSCs are germline-competent [4]. The methods for generating iPSCs have been improved since [5], particularly with the introduction of epigenetic factors [6] or epigenetic states altering compounds in conjunction with the Yamanaka factors. Neural progenitors [7] and many different neuron types [8,9,10] have been successfully generated from iPSCs. In 2010–2011, a direct conversion of mouse and human fibroblasts to “induced neuronal” (iN) cells with three factors, Ascl1, Brn2/Pou3f2 and Myt1l, was reported by Wernig and colleagues [11,12]. The same three factors could also convert human fibroblasts into specific neuron types such as dopaminergic neurons [13]. Direct lineage reprogramming using cells from a different germ layer, such as hepatocytes to neurons, was then shown to be feasible [14]. The generation of iN cells from other non-neuronal cell types have also been reported, with the techniques and procedures for doing so constantly being improved [15,16].

**Figure 1:**
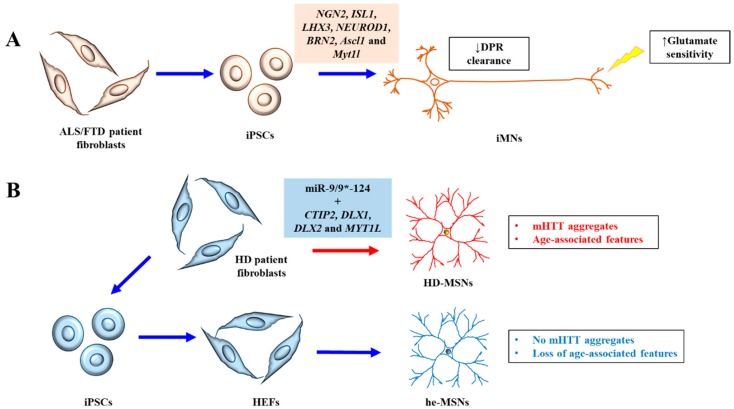
A schematic diagram illustrating the recent advances discussed. (**A**) Induced motor neurons (iMNs) generated from ALS/FTD patient iPSCs exhibiting increased sensitivity to glutamate and decreased clearance of dipeptide repeat (DPR) proteins. (**B**) Striatal medium spiny neurons directly induced from HD patient fibroblasts (HD-MSNs) exhibited clear HD neuronal phenotypes of mutant Huntingtin (mHTT) aggregates and age-associated features like DNA damage and oxidative stress, which is not seen in MSNs derived from human embryonic fibroblasts (HEFs) generated from patient-derived iPSCs (he-MSNs).

Both iPSCs and iNs have been used to model neurodegenerative diseases. An open-access collection of fibroblast lines from patients carrying mutations linked to neurological disease has in fact been created and deposited with the National Institute for Neurological Disorders and Stroke (NINDS) [17], which would facilitate research in this regard. The use of patient-derived iPSCs and iNs may offer novel insights into disease etiology not previously revealed by other proxies. This point is aptly demonstrated by two recent reports. In the first, iPSC-derived induced motor neurons (iMNs) from ALS/FTD patient with the open reading frame 72 in human chromosome 9 (*C9orf72*) repeat expansion revealed a previously unrecognized haploinsufficiency defect due to loss of C9ORF72 protein expression. This loss of function impaired endo-lysosomal protein trafficking and augmented glutamate as well as dipeptide repeat protein (DPR)-mediated neurotoxicity [18] (Figure 1A). In another study, striatal medium spinal neurons (MSNs) directly induced from HD patient fibroblasts showed mutant Huntingtin (mHTT) pathologies that are age-associated, which were not discernible in neurons differentiated indirectly via iPSCs from HD patients [19] (Figure 1B). These recent advances, together with other relevant new findings, are briefly reviewed and discussed below.

## 2. Modelling of Functional Haploinsufficiency Resulting from *C9orf72* Repeat Expansion

Amyotrophic lateral sclerosis (ALS) is classically known as a devastating motor neuron disease [20], while Frontotemporal dementia (FTD) is often presented as dementia resulting from neuron loss from the frontal or temporal lobes. Largely sporadic, ALS does present familial forms with mutations in genes like *SOD1* and the RNA binding proteins encoded by *TDP43* and *FUS* [21]. Both the clinical diseases, however, share common pathological features in terms of ubiquitinated neuronal inclusions of, for example TDP43, as well as common underlying genetic defects [22]. In particular, an intronic hexanucleotide (GGGGCC) repeat expansion in *C9orf72* [23,24] is now known to be the most common mutation for familial ALS and FTD [25]. This mutation is also found in sporadic forms of ALS. ALS/FTD are therefore clinically complex syndromes with a disease spectrum and a continuum of behavioral and cognitive pathological manifestations [26,27].

How does the repeat expansion in an intronic region of *C9orf72* cause disease? CNS neurons express high levels of C9ORF72, and the protein likely has important yet incompletely defined cellular functions in terms of autophagy [28,29,30,31] and vesicular traffic [32]. Much research on the neuropathology of *C9orf72* mutation has focused on the fact the repeat expansion generates neurotoxic species such as RNA/DNA G-quadruplexes [33], DPRs [34,35] and nuclear RNA foci, which sequesters important RNA-binding proteins and thus perturbs mRNA splicing and transport [36,37]. There is diminished C9ORF72 protein expression from the allele harboring a repeat extension via both direct and indirect mechanisms, but whether there is an associated haploinsufficiency phenotype has remained unclear. This uncertainty is fueled by the observations that complete *C9orf72* ablation in mice did not result in overt neurodegenerative phenotypes [38,39], neither do human patients with homozygosity for the *C9orf72* expansion suffer from a dramatically more severe disease. 

Shi and colleagues generated iMNs from ALS/FTD patient iPSCs via the co-expression of a number of proneural transcription factors (Ngn2, Isl1, Lhx3, NeuroD1, Brn2, Ascl1 and Myt1l). These iMNs express a number of spinal motor neuron markers, exhibit the same electrophysiological properties as motor neurons and could induce the contraction of co-cultured myotubes. The authors found that patient iMNs are consistently more sensitive to glutamate induced neuronal demise as well as death resulting from the withdrawal of neurotrophic factors, compared to control iMNs. Such a sensitivity was not observed with another neuron type, namely induced dopaminergic neurons (iDAs), derived from the iPSCs of *C9orf72* mutated patients. Importantly, reduced iMN survival can be rescued by the re-expression of C9ORF72 and the phenotype is recapitulated by one or both allele functionally deleting *C9orf72* using CRISPR/Cas9. 

What then, are the cellular consequences of reduced C9ORF72 levels in motor neurons? The authors noted that C9ORF72 is found in cytoplasmic puncta, co-localizing largely with the early endosome marker EEA1 (and may in fact physically interact with the latter), and more rarely with the late endosome marker LAMP1. Notably, patient iMNs deficient in C9ORF72 have a lower number of lysosomes. This reduction in lysosome number is corroborated by the observation that C9ORF72-deficient iMNs harbored packed clusters of mannose-6-phosphate receptor (M6PR)-bearing vesicles, a phenotype that is also rescued by the over-expression of C9ORF72. These findings attest to a critical role for C9ORF72 in cellular membrane traffic leading to lysosome biogenesis in the iMNs. What caused the enhanced sensitivity to glutamate toxicity? Interestingly, the C9ORF72-deficient iMNs have elevated levels of glutamate receptor subunits (both AMPA receptor (GluR1 subunit) and NMDA receptors (NR1 subunit)) on their neurites and dendritic spines. The same observation was made for spinal motor neurons from mice with a conditional *C9orf72* deletion in CNS neurons (Nestin-Cre-Stop-Flox-*C9ORF72*), while postsynaptic density of the motor cortices of *C9orf72*-mutated patients also have higher levels of GluR1 and NR1.

Another important finding from Shi et al.’s paper is that C9ORF72 deficiency sensitized iMNs to not only glutamate, but also a key neurotoxic product of the repeat expansion, namely the aggregation-prone DPRs, generated via repeat-associated non-AUG (RAN) translation. In fact, C9ORF72 deficiency impaired the cells’ ability to clear DPR aggregates, which is unrelated to the cell’s overall proteostatic capacity as the clearance of other types of aggregates were not affected. Is the DPR aggregate clearance effect related to the defect in membrane traffic resulting from C9ORF72 deficiency? A screen for small molecules that might rescue the patient iMN survival defect identified a candidate which is an inhibitor of FYVE finger-containing phosphoinositide kinase (PYKFYVE) that converts phosphatidylinositol 3-phosphate (PI3P) into phosphatidylinositol (3,5)-bisphosphate (PIP2). PI3P is a key driver of endosomal maturation and autophagosome lysosomal fusion and it also functions at the initiation part of autophagosome formation. Amplimod, a structurally different PYKFYVE inhibitor, also rescued the deficiency in patient iMN survival, but did not improve the survival of control iMNs. Interestingly, a constitutively active form of RAB5 (which recruits PI3-kinase and promote PI3P synthesis on membranes) also selectively promoted patient iMN survival. That PYKFYVE inhibition could be useful in an animal disease model is illustrated by Apilimond’s rescue of NMDA-induced neurodegeneration in C9ORF72-deficient mice.

On the whole, the findings of Shi et al. have several important implications, all of which are intricately linked. The first is a strengthening of the notion that C9ORF72 plays an important role in endosome maturation and endo-lysosomal traffic. This notion was initially suggested by previous work implicating C9ORF72’s possible functional interaction with endosomal and exocytic Rabs [30,40,41] and is also in line with the fact that membrane trafficking components are amongst those known to be mutated in ALS [21]. In the current work, this notion is affirmed by the defective M6PR trafficking and potentially also by the increased glutamate receptor expression at the postsynaptic surfaces. The latter finding is corroborated by another recent report by Selvaraj and colleagues, which also showed an increase in the AMPA receptor subunit in motor neurons generated from ALS patient iPSCs, which could be abolished by CRISPR/Cas9-mediated correction of the *C9ORF72* repeat expansion [42]. Selvaraj et al. have also made similarly relevant observations with post-mortem ALS patient samples. Interestingly, the hypersensitivity of C9ORF72 mutant motor neurons is not recapitulated in iPSC-derived *cortical neurons*, which attests to the specific vulnerability of motor neurons in this regard. However, as M6PR trafficking and AMPA receptor trafficking occur via different compartments and itineraries, involving exocytosis and endocytosis as well as the recycling pathways, the exact membrane trafficking steps that C9ORF72 participates in and the critically of its involvement in these steps would need further exploration.

Secondly, it now appears that allelic *C9ORF72* repeat extension does result in the haploinsufficiency of protein expression that contributes to disease. The complex disease phenotype in ALS/FTD caused by C9ORF72 repeat extension is therefore most likely driven by both gain-of-function and loss-of-function mechanisms, with the latter potentially augmenting the former. The findings also provided a potential (albeit partial) explanation of why C9ORF72 knockout mice have no obvious neurodegenerative phenotype, as these do not generate DPRs that would sensitized motor neurons to stress and death. What is not yet explained is why C9ORF72 deficiency impacts motor neurons rather specifically (or more severely), both in terms of trafficking defects and glutamate receptor surface expression. A hint of the latter could come from further understanding of how C9ORF72 fits into PYKFYVE and RAB5’s reciprocal roles in modulating PI3P and membrane traffic, and how C9ORF72’s involvement might differ between neuron types.

The sporadic forms of ALS and FTD are both aging-associated. The question of how aging may enhance disease phenotype manifestation in ALS/FTD models is not addressed in Shi et al. [18]. Whether age-associated phenotypes or signatures could be effectively addressed by iPSC-derived neurons has in fact been questioned, as it is conceivable that the process of reprogramming towards pluripotency in the making of iPSCs could erase aging-associated epigenetic marks and consequently alter gene expression profiles corresponding to the aged disease state. In a related recent report, Zhang’s group has indeed shown that iPSC reprogramming resets the aging-associated features of old fibroblasts, as well as the motor neurons derived from these [43]. The authors used a set of transcription factors termed NSIL (NGN2, SOX11, ISL1, and LHX3) that allowed the derivation of motor neurons from either iPSC-derived neural progenitor cells (termed iPSC-MN), or directly from fibroblasts (termed Fib-iMN) [44]. Interestingly, the Fib-iMNs retained aging-associated features that are dependent on the age of the fibroblasts’ donor. Fib-iMNs from old donors had a higher number of cells exhibiting γH2AX foci (a marker of DNA damage) and senescence-associated β-galactosidase staining when compared to Fib-iMNs from young donors. In contrast, there are no detectable differences in terms of aging-associated markers between different iPSC-MNs derived from the fibroblasts of different age donors, thus attesting to the notion of aging feature erasure by the iPSC reprogramming step. Maintaining the aging-associated features would be very useful for the modelling of chronic, long and drawn-out neurodegenerative diseases, such as HD. This is illustrated by the case below.

## 3. Modelling of Striatal Neuron Pathology by Direct Conversion from HD Patient Fibroblasts

HD, also known as Huntington’s chorea, presents a typical symptom of involuntary jerky movements [45]. However, the full disease state may be preceded by general problems in movement coordination and unsteady gait, accompanied by psychiatric disturbances and cognitive decline, with the latter eventually progressing into dementia. Clear disease onset typically occur in midlife and the patient’s condition gradually worsens over the course of 15–20 years towards eventual fatality. HD is caused by a monogenic defect, namely a CAG repeat extension at the N-terminus of the gene encoding Huntingtin (*HTT*) [45]. The CAG repeat translates to an abnormally long (typically >36) polyglutamine (polyQ) stretch at HTT’s N-terminus. HTT is a multifunctional scaffolding protein involved in a myriad of cellular processes, which include gene transcription, cell signaling, axonal transport and synaptic transmission [46]. In the brain, the neuropathology elicited by mutant HTT (mHTT) primarily affects striatal neurons, with a loss of medium spiny projection neurons (MSNs) that project to the globus pallidus and the substantia nigra, as well as neurons that project from the cortex to the striatum. Neuronal pathology could be due to a loss of mHTT function negatively impacting brain-derived nerve growth factor (BDNF) signaling [47,48], but it is largely attributed to the toxic gain-of-function by mHTT and its proteolytic products. These products form nuclear and cytoplasmic aggregates that perturb nuclear transcription and nuclear-cytoplasmic dynamics [49,50]. Perturbations of cytoplasmic and axonal transport processes also contribute to neuronal pathology and synaptic dysfunction [46].

The long drawn out disease course of HD makes age a particularly important factor to be appropriately captured by its model. HD modelling with patient iPSC has been attempted. However, neurons derived from HD patient iPSCs often do not readily exhibit clear neuropathological manifestations and compromised survival unless further subjected to excitotoxic or oxidative stresses. These iPSC-derived neurons also do not clearly exhibit a pathological hallmark of HD, namely the mHTT aggregates, with these becoming prominent only after prolonged culture or treatment with proteasome inhibitors [9,51,52]. One possible underlying reason for this rather subdued pathological phenotype manifestation in HD patient iPSC-derived neurons could be the intervening reprogramming process. In a recent report, Yoo’s laboratory showed that age-associated disease phenotypes of HD are better recapitulated by striatal MSNs directly induced from patient fibroblasts [19,53].

In generating MSNs directly induced from the fibroblasts of HD patients (HD-MSN) and healthy controls, the authors used a combination of brain-enriched microRNAs (miR-9/9* and miR-124), which reconfigures chromatin accessibility [54], together with the transcription factors CTIP2, DLX1, DLX2 and MYTL1 [19,53]. The HD-MSNs generated had increased excitability and firing pattern complexity compared to control MSNs that is reflective of the HD disease phenotype. These also exhibited differential gene expressions that are either previously known to be altered in human HD brains (such as the upregulation of the matrix metalloprotease gene *MMP9* and downregulation of the BDNF receptor gene *TRKB*), or could be correlated with neuronal excitability and neuronal survival (such as upregulation of the α-synuclein gene (*SNCA*) and that encoding the pro-survival transcription factor *SP9*). Importantly, HD-MSNs exhibited obvious mHTT aggregates, which was not the case for either the fibroblasts they were derived from, or the control MSNs. Some of these aggregates also appeared to have been incorporated within the autophagy marker Light chain 3-II (LC3-II) positive autophagosomes.

Is the more prominent disease phenotype exhibited by the HD-MSNs due to the direct derivation? The authors directly addressed the question as to whether iPSC reprogramming had influenced the mHTT aggregation phenotype. iPSCs from HD patient fibroblasts were derived and then differentiated back into human embryonic fibroblasts (HEFs). Notably, the MSNs subsequently induced from these HEFs (termed human embryonic MSNs, or he-MSNs) exhibited little or no mHTT aggregates. What cellular aspects underlie the difference between the adult HD-fibroblast-derived HD-MSNs and the embryonic fibroblast-derived he-MSNs in terms of mHTT aggregate formation? When GFP-tagged polyglutamine repeats (GFP-74Q) were overexpressed in the respective fibroblasts, the adult fibroblasts formed inclusion bodies with GFP-74Q aggregates much more readily than the HEFs. A transcript analysis of a panel of genes associated with the ubiquitin-proteasome system (UPS) indicated that eight such genes were consistently upregulated in HEFs. These included *HSF1*, which encodes Heat shock factor 1, a key regulator for protein homeostasis during stress [55]. iPSCs are known to have an elevated proteostasis capacity and proteasome activity compared to fibroblasts or neurons [56]. In assessing the functionality of the proteasome, the authors found that proteostasis was significantly diminished in HD-MSNs compared to heMSNs, with the latter retaining a proteasome activity that is comparable to that of the iPSCs. Furthermore, by gene profiling analysis of young versus old fibroblasts and MSNs, it is clear that MSNs derived from older individuals have a much higher number of downregulated UPS-related genes. A difference in proteostatic capacity, manifested at least partly through expression level changes of UPS-associated genes, therefore underlies the blunted mHTT aggregation phenotype in iPSC-derived MSNs.

The HD-MSNs are, on the whole, a fairly sensitive model for HD pathology resulting from mHTT toxicity. These neurons exhibited DNA damage and spontaneous neurodegeneration that is dependent on the mHTT, which could be alleviated by shRNA-mediated silencing of HTT. They also exhibited high levels of mitochondrial dysfunction and the consequential oxidative stress, metabolic deficiency and an upregulation of mitophagy. That the toxic effect of mHTT is dependent on the neuronal cell type was demonstrated by the fact that *cortical neurons* derived directly from HD fibroblasts, despite also presenting a high level of mHTT aggregation, exhibited lower levels of DNA damage and degeneration compared to the HD-MSNs. Furthermore, a clear value of HD-MSN lies in its ability to model aspects of HD neuronal pathologies that are age-dependent. The authors showed that MSNs derived from patients in the presymptomatic stage of the disease, despite also exhibiting mHTT aggregates, were significantly less vulnerable to mHTT-induced toxicity, as these showed significant lower levels of oxidative DNA damage and neurodegeneration. 

## 4. New and Future Perspectives

The recent findings discussed above have attested to how patient-derived iPSCs and iNs have shed new light on the cellular etiology of neurodegenerative diseases. From the perspective of ALS/FTD, it is now clearer that *C9orf72* repeat expansions that diminished protein expression likely contributed in a loss-of-function manner to the disease phenotype. This contribution takes at least the form of heightened glutamate excitotoxic response and impaired clearance of DRPs [18], with a likely underlying mechanism being a deficiency in membrane trafficking processes. With regard to the latter, much remains to be learned. Immediate questions to pursue would include clearly defining the involvement of C9ORF72 in glutamate receptor transport and DPR clearance. Regarding DPR clearance, it is still unclear whether the defect has more to do with impaired autophagy, in which C9ORF72 is already known to play certain roles [28,29,30,31,41,57], or other less well-defined proteostasis pathways.

While the usefulness of iPSCs in the derivation of diseased neurons is apparent, the findings that the neurons directly induced from patient fibroblasts could better retain age-associated disease features represent an important further advance in neurodegenerative disease modelling [19,43]. That young neurons are better able to withstand the toxicity of proteins mutated in familial forms of neurodegenerative diseases (such as mHTT. DRP, α-synuclein and other polyglutamine expansion mutants) than older neurons, although intuitively obvious, was difficult to probe mechanistically. The efficient conversion of patient fibroblasts to different neuron types would therefore allow longitudinal sampling of fibroblasts at different ages from the same individuals to facilitate the accurate comparative analysis of the induced neurons. Notably, the ALS/FTD patient iPSC-derived iMNs and the directly induced HD patient MSNs both showed that these recapitulated the neuronal cell-type-specific susceptibility known for the respective diseases. The derived neurons therefore hold great promise for the further elucidation of the molecular pathological mechanism underlying these diseases.

It is worth noting that while iPSCs have their limitations, particularly pertaining to the retention of age-associated phenotypes, these cells could nonetheless be more versatile and wieldy compared to iNs. Immortal and self-renewing, iPSCs have the advantage of yield and are amplifiable, as well as the potential for the subsequent generation of multiple different neuronal subtypes. iNs are terminally differentiated and can only be reproduced with the availability of patient-derived somatic cells. Also, while a one-step direct conversion from fibroblasts to neurons would be intuitively faster than an indirect derivation from iPSC (or NPCs from iPSC), the purity and yield may be conceivably lower. In this regard, however, the recent advances discussed above have all come with good conversion yields. With the Yoo lab protocol for HD-MSNs, the authors achieved a reasonably high yield (>85% of converted cells expressing neuronal markers MAP2, NeuN and βIII-tubulin, with a majority of MAP2-positive cells being GABAergic, and with 70% of these expressed the striatal neuron marker DARPP32 [53]). In the Zhang lab NSIL protocol for generating Fib-iMNs, HB9-positive and ChAT-positive motor neurons were generated with comparable efficiency from both fibroblasts and iPSC-derived NPCs [43]. Methods for generating the GABAergic medium spiny neurons and the cholinergic motor neurons directly from patient fibroblasts are therefore now established.

Finally, better disease models and a clearer understanding of pathological mechanisms would facilitate the discovery of new therapeutics. In this regard, Shi et al. has shown that PYKFYVE inhibitors may offer potential novel therapeutic options for ALS/FTD. The field is of course in desperate need for new drug options [58], as riluzole, first approved 20 years ago by the FDA, has remained the only approved drug until the recent approval of edavarone [59]. On the other hand, any HD treatment available is only symptomatic against the neurological and neuropsychiatric presentations. A better understanding of age-associated factors in HD progression may open doors towards finding early interventions at the prodromal stage of the disease that could more effectively delay disease onset and better suppress neurological symptoms.
